# Role of Vitamin D in Uremic Vascular Calcification

**DOI:** 10.1155/2017/2803579

**Published:** 2017-02-12

**Authors:** Yi-Chou Hou, Wen-Chih Liu, Cai-Mei Zheng, Jing-Quan Zheng, Tzung-Hai Yen, Kuo-Cheng Lu

**Affiliations:** ^1^Division of Nephrology, Department of Internal Medicine, Cardinal Tien Hospital, School of Medicine, Fu-Jen Catholic University, New Taipei City, Taiwan; ^2^Graduate Institute of Clinical Medicine, College of Medicine, Taipei Medical University, Taipei, Taiwan; ^3^Division of Nephrology, Department of Internal Medicine, Yonghe Cardinal Tien Hospital, New Taipei City, Taiwan; ^4^Department of Internal Medicine, School of Medicine, College of Medicine, Taipei Medical University, Taipei, Taiwan; ^5^Division of Nephrology, Department of Internal Medicine, Shuang Ho Hospital, Taipei Medical University, New Taipei City, Taiwan; ^6^Division of Critical Care Medicine, Department of Emergency Medicine-Critical Care Medicine (EM-CCM), Shuang Ho Hospital, Taipei Medical University, New Taipei City, Taiwan; ^7^Department of Nephrology and Division of Clinical Toxicology and Toxicology Laboratory, Chang Gung Memorial Hospital, Linkou Medical Center, Taoyuan, Taiwan; ^8^Division of Nephrology, Department of Medicine, Tri-Service General Hospital, National Defense Medical Center, Taipei, Taiwan

## Abstract

The risk of cardiovascular death is 10 times higher in patients with CKD (chronic kidney disease) than in those without CKD. Vascular calcification, common in patients with CKD, is a predictor of cardiovascular mortality. Vitamin D deficiency, another complication of CKD, is associated with vascular calcification in patients with CKD. GFR decline, proteinuria, tubulointerstitial injury, and the therapeutic dose of active form vitamin D aggravate vitamin D deficiency and reduce its pleiotropic effect on the cardiovascular system. Vitamin D supplement for CKD patients provides a protective role in vascular calcification on the endothelium by (1) renin-angiotensin-aldosterone system inactivation, (2) alleviating insulin resistance, (3) reduction of cholesterol and inhibition of foam cell and cholesterol efflux in macrophages, and (4) modulating vascular regeneration. For the arterial calcification, vitamin D supplement provides adjunctive role in regressing proteinuria, reverse renal osteodystrophy, and restoring calcification inhibitors. Recently, adventitial progenitor cell has been linked to be involved in the vascular calcification. Vitamin D may provide a role in modulating adventitial progenitor cells. In summary, vitamin D supplement may provide an ancillary role for ameliorating uremic vascular calcification.

## 1. Introduction

Chronic kidney disease (CKD), a complex and common disease, has multiple complications with severe impacts. The risk of cardiovascular death is 10 times higher in patients with CKD than in those without CKD. This risk is even up to 100-fold higher in young patients with CKD than in those without CKD [1]. Progressive decline in the estimated glomerular filtration rate (eGFR) is associated with an increased risk of major cardiovascular events and all-cause mortality [2]. Moreover, vascular calcification, common in patients with CKD, is a predictor of cardiovascular mortality. Vascular calcification in CKD involves two pathologies: atherosclerosis and arteriosclerosis [3]. In patients with CKD, the dysregulation of calcium and phosphate metabolism induces vascular smooth muscle calcification, and CKD complications, such as renin-angiotensin-aldosterone system (RAAS) activation or insulin resistance, induce endothelial dysfunction and atherosclerosis. These pathologies coexist during CKD progression and exacerbate vascular calcification.

Vitamin D deficiency, another complication of CKD, is associated with vascular calcification in patients with CKD [4]. GFR decline, proteinuria, or tubular dysfunction aggravates vitamin D deficiency and reduces its pleiotropic effect on the cardiovascular system. This review assessed the role of vitamin D in uremic vascular calcification.

## 2. Vitamin D Metabolism

Vitamin D is synthesized in the human skin or obtained from the diet. 7-Dehydrocholesterol in the skin is converted to previtamin D3 upon exposure to ultraviolet B radiation. Vitamin D from the diet, vitamin D2 (ergocalciferol) or animal vitamin D3 (cholecalciferol), is identical to the skin-synthesized vitamin D3. The enzyme vitamin D 25-hydroxylase metabolizes ergocalciferol and cholecalciferol in the liver and converts them to the 25(OH)D forms of 25(OH)D2 and 25(OH)D3, respectively. 25(OH)D combined with vitamin D-binding protein (DBP) is delivered to the kidneys and filtered through the glomerulus [5]. The delivery of the 25(OH)D-DBP compound to the proximal tubular cells is facilitated by megalin receptor-mediated endocytosis [6]. Furthermore, 25(OH)D is converted to its active form, calcitriol, by 1-*α*-hydroxylase and transported by intracellular DBP3; thus, 1,25(OH)2D or 25(OH)D reenters the circulation. Vitamin D receptor analogues (VDRAs), such as calcitriol, paricalcitol, and maxacalcitriol, directly act on the VDR [7].

Vitamin D has pleiotropic effects on immunity, the cardiovascular system, bone, the pancreas, the breast, and parathyroid hormone (PTH). In patients with CKD, vitamin D deficiency is common and is associated with overall and cardiovascular mortality. Vascular calcification is a crucial contributor to mortality in CKD and end-stage renal disease (ESRD). Vitamin D supplements increase the survival of patients with CKD [8, 9]. Therefore, our review focused on the effects of vitamin D on vascular calcification in patients with CKD.

## 3. Vitamin D Deficiency in Patients with CKD: Mechanism (Figure 1)

Recent observations have demonstrated that kidney disease seems to be associated with a high incidence of vitamin D insufficiency or deficiency [10]. Studies by González et al. demonstrated that 25-hydroxyvitamin D values are <30 ng/mL, believed to be the lower limit of normal, in the majority of patients with CKD [11]. Patients who are severely proteinuric have the lowest values. These investigators have shown that virtually all of the secondary hyperparathyroidism that occurs in the course of CKD is associated with 25-hydroxyvitamin D values that are <30 ng/mL. It is interesting to note that, in this patient group, there is a positive relationship between 25-hydroxyvitamin D levels and 1,25-dihydroxyvitamin D levels, in contrast to what is seen in normal individuals. Thus, when 25-hydroxyvitamin D levels are increased by therapy, one would anticipate an increase in the levels in the 1,25-dihydroxyvitamin D. It is not clear whether this is a contribution of renal 1-*α*-hydroxylase or the 1-*α*-hydroxylase at extrarenal sites; however, because of the association of low levels of 25-hydroxyvitamin D with hyperparathyroidism in the course of CKD, it is recommended that, in patients with CKD, if hyperparathyroidism is detected, then 25-hydroxyvitamin D should be measured, and if found to be <30 ng/mL, then the initial step in the therapy should be to try to correct this abnormality, as the first step in the control of hyperparathyroidism. Proteinuria, tubulointerstitial injury, GFR loss, and reduction of hepatic cytochrome p450 by pharmacologic dosage of active vitamin D are the possible mechanisms inducing vitamin D deficiency [12].

### 3.1. Vitamin D Deficiency because of Proteinuria

Vitamin D deficiency is common in patients with proteinuria. In nephrotic syndrome, vitamin D deficiency is common, and it predicted the remission of nephrotic syndrome in a case-control study [13]. In patients with proteinuria, such as those with diabetic nephropathy, the binding of 25(OH)D to the megalin receptor is limited; thus, fewer receptors are available for 25(OH)D-DBP reabsorption. In addition, consequent to proteinuria, proximal tubular cells are damaged; hence, fewer megalin receptors are available [14].

### 3.2. Vitamin D Deficiency because of GFR Decline

The prevalence of vitamin D deficiency is high in patients with an impaired GFR. An impaired GFR is predictive of vitamin D deficiency [15]. GFR decline limits the delivery of 25(OH)D to the renal tubules. The decreased renal uptake of 25(OH)D limits the formation of calcitriol. Because of GFR decline, the phosphaturic hormone fibroblast growth factor (FGF) 23 is synthesized from osteocytes. FGF23 inhibits the 1-*α*-hydroxylase activity in the renal proximal tubule to reduce 1,25(OH)2D production and stimulates 24-hydroxylase to produce 24,25(OH)2D [16]. In contrast to FGF23, PTH, another phosphaturic hormone, increases the 1-*α*-hydroxylase activity. However, the function of PTH is reduced by the retention of uremic toxins and metabolic acidosis because of GFR decline and the uncoupling of the PTH receptor-protein kinase A axis [17, 18]. The decrease in klotho protein in the blood is an early event in CKD and is progressively reduced along with the decline of renal function. Low klotho partially induces FGF23 resistance, causing an initial compensatory increase in blood FGF23 to maintain P homeostasis. The increase in FGF23 decreases vitamin D levels and is followed by elevation of PTH. Hyperphosphatemia is relatively late event in advanced CKD [19]. Thus we call the FGF/PTH as the killers, but the phosphate as the chief instigator and vit-D as a victim in the development of CKD-MBD.

### 3.3. Vitamin D Deficiency because of Tubulointerstitial Damage

Renal tubular epithelial cells possess 1-*α*-hydroxylase, which converts 25(OH)D to 1,25(OH)2D, and 24-hydroxylase, which converts 25(OH)D to 24,25(OH)2D. Serum 1,25(OH)2D reduces the 1-*α*-hydroxylase activity in renal cells and promotes 24-hydroxylase gene activity for enhancing 1,25(OH)2D inactivation [20]. CKD progression in addition to tubulointerstitial damage reduced the activity of 1-*α*-hydroxylase and 25-hydroxylase. In addition, in uremic rats, indoxyl sulfate upregulated nuclear factor-*κ*B expression in renal tubular cells and subsequently activated 24-hydroxylase [21, 22]. Increased 24-hydroxylase and decreased 1-*α*-hydroxylase activities cause a prominent reduction of endogenous 25(OH)D and 1,25(OH)2D products, thus increasing their decay. The severity of vitamin D deficiency increases with the progression of tubulointerstitial damage.

### 3.4. Therapeutic 1,25(OH)_2_D_3_ Usage

In CKD patients with secondary hyperparathyroidism (SHPT), vitamin D, particularly the active form of vitamin D, inhibits the parathyroid gland. Nigwekar et al. addressed the question of calcidiol deficiency, one potential factor that was not mentioned is a reduction in hepatic conversion of calciferol into calcidiol, as shown in a chronic kidney failure rat model [23]. This reduction is secondary to downregulation of the major cytochrome P450 isoforms involved in 25-hydroxylation of vitamin D in rats. Furthermore, the mechanism underlying decreased cytochrome P450 activity seems to be related to secondary hyperparathyroidism. This mechanism also could explain the poor response obtained when treating some patients with CKD with 1*α*-hydroxyvitamin D [24]. The normal serum physiological 1,25(OH)2D concentration is approximately 20–30 pg/mL (50–75 pmol/L) [25]. The therapeutic dose of 1,25(OH)2D (usual dose in micrograms) is crucial for treating SHPT in patients with CKD. However, 1,25(OH)2D is the end product of the vitamin D pathway and inhibits 1-*α*-hydroxylase and 25-hydroxylase through feedback inhibition. A therapeutic dose of 1,25(OH)2D may downregulate 25(OH)D levels, thus reducing 25(OH)D availability in extrarenal tissues and organs and increasing 25(OH)D deficiency [26].

## 4. The Mechanisms of Cardiovascular Calcification in CKD

Vascular calcification is a prominent feature of arterial disease in CKD and may have an impact on cardiovascular mortality through modulating both arteriosclerosis (arterial stiffening) and atherosclerosis. According to the anatomical site, vascular calcification can be divided into three categories: atherosclerosis, arteriosclerosis, and cardiac valve calcification. During the progression of CKD, atherosclerosis and arteriosclerosis occur simultaneously because of mixing effects by hyperparathyroidism, renal bone dystrophy, metabolic syndrome, hypertension, retention of uremic toxin, and transformation of adventitial progenitor cells. The following are the mechanisms.

### 4.1. Traditional Concept of Mechanisms of Vascular Calcification in CKD: On Endothelium [27]

Atherosclerosis involves the intima layer of arterial vessels. Lipid-laden plaque within the tunica intima is a hallmark of atherosclerosis, and atheromas are mainly composed of macrophages with high low-density lipoprotein (LDL) and triglyceride levels. In addition to dyslipidemia, oxidative stress and chronic inflammation contribute to endothelial dysfunction and subsequent atherosclerosis. In patients with diabetes mellitus with preserved renal function, microalbuminuria, an indicator of endothelial dysfunction, predicts the presence and progression of coronary arterial calcification [28, 29]. Oxidative stress induces endothelial dysfunction. Oxidative stress is triggered by risk factors contributing to endothelial shearing stress change, such as RAAS aldosterone activation and hyperfiltration in diabetes mellitus, and chronic inflammation exacerbates insulin resistance or metabolic syndrome [30, 31]. With deteriorating renal function, the accumulation of indoxyl sulfate damages the endothelial cells by enhancing monocyte adhesion, thus increasing endothelial oxidative stress induced stimulated by inflammatory cytokines, and inhibits endothelial progenitor cell-associated neovascularization [32]. During early-stage CKD, the dysregulation of the calcium-phosphate balance influences endothelial injury and subsequent endothelial dysfunction. However, the disruption of endothelial-derived relaxing factors may signal an early stage in atherosclerosis. Hyperlipidemia, hypertension, metabolic syndrome, protein bound uremic toxins (indoxy sulfate/p-cresol sulfate), and CKD are the major causes of endothelial injury, partly through increase of inflammation or oxidative stress. Major cell players are endothelial cells (or valve interstitial cells; VICs), leukocytes, and intimal smooth muscle cells (SMC). Focal calcification within atherosclerotic plaques is due to both active (osteogenic) and passive (cellular necrosis) processes. The phenotypic osteocyte in calcified vessels/valves may secrete Wnt inhibitors, which may fight back inhibition of bone formation.

### 4.2. Bone Turnover and Vascular Calcification [33]

CKD progression results in less vitality of bones in patients with CKD than in normal people. Thus, low bone turnover is an innate characteristic of CKD. High PTH serum levels stimulate indolent bone cells and lead to high-turnover bone disease, with the characteristics of relatively higher bone resorption than bone formation [34]. The high bone turnover status in SHPT can induce an increase in bone demineralization, which increases calcium and inorganic phosphate release from the bones into circulation. Most CKD patients developed high PTH levels after stage 3 of CKD. Patients may present prominent soft tissue calcification and/or vascular clarification [35]. In low or high bone turnover, serum calcium and phosphate concentrations increase and excessive calcium and phosphate precipitate in the vessels [36–38]. In overtreatment of CKD patients with Ca-salts, VDRA, aluminum, or parathyroidectomy may cause them to develop low turnover bone disorders and low serum PTH levels. In patients with low bone turnover status, the decreased bone mineralization makes it difficult for calcium and inorganic phosphate to enter into bone, resulting in increased serum calcium and inorganic phosphate. Thus, patients may present with prominent vascular calcification. Both high and low bone turnover disorders are characterized by a relatively higher degree of bone resorption than bone formation, which may contribute to the elevated serum calcium and inorganic phosphate levels, and aggravate vascular calcification/ossification. Therefore, correcting the high or low turnover status of bones is crucial for alleviating vascular calcification.

### 4.3. PTH and Vascular Calcification

The elevation of FGF23 increases the degradation of 25(OH)D by enhancing the activity of 24-hydroxylase and inhibition of 1-*α*-hydroxylase. The expression of klotho from distal renal tubules decreases with decreasing 25(OH)D. In addition, indoxyl sulfate prevents the calcitriol-induced inhibition of parathyroid cell proliferation. PTH acts on PTH receptors on osteoblasts and drives the proliferation of hematopoietic stem-progenitor cells (HSPCs) in the bone marrow either directly [39] or through the stimulation of granulocyte-colony stimulating factor, which consequently, through osteoblast loss and reduced CXCL12 expression by the cells inside the niche, fosters HSPC transmigration into the vascular sinuses [40, 41]. The circulating CD34 progenitor cells and CD34-positive vascular endothelial growth factor receptor-2-positive endothelial progenitor cells, which are correlated with vascular calcification, worsen the endothelial dysfunction [42]. Previously, smooth muscle cells, mesenchymal stem cells, and pericytes were considered to be the major precursors of ectopic chondrogenic cells in calcified vessels. In recent studies, endothelial cells have been demonstrated to participate in tissue calcification by providing osteochondrogenic cells via the endothelial-to-mesenchymal transition. Recent animal study showed elevated PTH induces endothelial to chondrogenic transition in aortic endothelial cells [43]. It also showed that cinacalcet ameliorates aortic calcification in uremic rats via suppression of endothelial-to-mesenchymal transition [44]. These data showed high PTH levels may contribute to the development of the intima (endothelial) calcification (atherosclerosis) other than the phosphate induced medial layer calcification.

### 4.4. Hyperphosphatemia and Vascular Calcification

In early CKD, compensatory mechanisms mediated by FGF23 maintain phosphaturia at a sufficient level. With increasing renal function impairment, phosphate retention induces compensating elevation of the phosphaturic hormones FGF23 and PTH. Phosphate retention occurs very early in the course of CKD, and it contributes to the genesis of SHPT [45]. In dialysis patients with calciphylaxis, hyperphosphatemia and calcium × phosphate product, but not PTH, were found to be risk factors in case-control studies [46, 47]. Hyperphosphatemia stimulates endothelial cells to release microparticles, which reduce the secretion of annexin II, reduce angiogenesis, increase the production of reactive oxygen species, and enhance inflammation, resulting in apoptosis of the endothelial cells [48]. Serum phosphate influences the endothelial response; inorganic phosphate induces endothelial dysfunction by producing oxidative stress and reducing nitric oxide production, by a higher magnitude than does indoxyl sulfate [49]. In CKD, abnormal mineral metabolism, predominantly hyperphosphatemia and hypercalcemia, facilitates the progression of the active process of osteogenesis in vascular smooth muscle cells (VSMC) resulting in arteriosclerosis calcification. VSMCs cultured in higher phosphorus concentrations express genes as markers of osteoblasts and induce both calcification in extracellular tissues and osteochondrogenesis [50]. Hyperphosphatemia plays an important role in the development of vascular calcification.

### 4.5. FGF23/Klotho and Vascular Calcification

FGF23, secreted by osteocytes, induces left ventricle hypertrophy. However, studies have reported direct effects of FGF-23 and klotho on vascular calcification. Recent clinical and observational data suggest that FGF23 is linked to cardiovascular mortality as well as subclinical indices of cardiovascular pathology such as left ventricular hypertrophy, vascular calcification, and endothelial dysfunction [51]. In patients at various CKD stages, plasma FGF23 is an independent biomarker of vascular calcification [52]. In observation studies, FGF-23 seemingly exerted an anticalcification effect. FGF23-knockout mice show severe vascular calcification in addition to hyperphosphatemia. FGF23 mutation is associated with ectopic calcification [53]. However, in animal study, these results demonstrate that FGF23-klotho signaling is absent in mouse arteries and that the vascular response was unaffected by FGF23 treatment [54]. However, when the effects of FGF23 have been blocked with monoclonal anti-FGF23 antibodies in an experimental animal model of CKD, even if hyperparathyroidism was better controlled, the net result was a net increase in animal mortality [55], and these data cast some doubt on the putative direct pathogenic effect of FGF23. In end-staging kidney disease, impaired compensatory mechanisms by further downregulation of klotho promote osteochondrocytic differentiation of VSMCs through phosphate hoarding and increased transcription factors. On the other hand, klotho protein expressed in VSMCs suppresses osteochondrocytic differentiation with inhibition of phosphate uptake. Both renal and vascular klotho protect VSMCs against vascular calcification. In klotho-deficient vessels, the deficiency is associated with vascular calcification, and it mediates the resistance of FGF23 [56]. From the evidence above, the interaction between FGF23 and klotho on vascular calcification needs further investigation.

### 4.6. Decrease of Calcification Inhibitors and Vascular Calcification

Renal function impairment reduces endogenous calcification inhibitors, such as fetuin-A, matrix *γ*-carboxyglutamic acid protein (MGP), pyrophosphate, osteoprotegerin, and bone morphogenetic protein. Fetuin-A, a hepatic secreting protein, is a crucial inhibitor of extraskeletal calcification that exerts its action by inhibiting the de novo formation and precipitation of calcium phosphate [57]. Low fetuin-A concentrations are associated with high coronary arterial calcification scores in patients undergoing HD [58]. Fetuin-A is synthesized in the liver as a negative acute-phase protein. Low fetuin-A reflects malnutrition in patients undergoing HD, and low serum fetuin-A is associated with more severe vascular calcification and subsequent cardiovascular mortality [22]. MGP, synthesized by VSMCs [59], is observed at the interface between normal tissues and the mineralized lesions of calcified arteries in patients with CKD or diabetes mellitus. Pyrophosphate, degraded by alkaline phosphatase on the bone lining cells, serves as inhibitors of vascular calcification. Bisphosphate is resistant to alkaline phosphatase degradation, and it helps to ameliorate calcification. However, it increase serum iPTH after lowering serum calcium concentration [27, 60, 61]. Studies have reported that the vitamin K-dependent *γ*-carboxylation of glutamate residues is mandatory for MGP's ability to chelate minerals and inhibit calcification [62]. In rats, uremia impairs vitamin K recycling by reducing the *γ*-carboxylase activity [63]. In patients undergoing maintenance HD, low vitamin K is common because of low protein intake and high energy wasting; vitamin K antagonists, such as warfarin, are strongly associated with arterial calcification [64–66]. Exposure to vitamin K antagonists has been recognized as a predictor of vascular calcification in patients undergoing maintenance HD [23, 67].

### 4.7. Adventitial Cell and Vascular Calcification

Mesenchymal stem cell-like cells are present in the vascular wall, particularly in the inner layer of the tunica adventitia. After intimal injury, intimal endothelial progenitor cells are present in the reendothelialization area. Submural muscular progenitor cells provide rapid replacement after insult to the tunica media according to the outside-in paradigm [68]. Soluble mediators released from adventitial progenitor macrophages, such as TGF-*β* and platelet-derived growth factor, activate the sonic hedgehog (Hh) signal and subsequently smooth muscle transformation from SMCs through Gli1. Perivascular Gli1^+^ progenitors are key contributors to injury-induced organ fibrosis [69]. Gli1^+^ cells located in the arterial adventitia are progenitors of VSMCs and contribute to neointima formation and repair after acute injury to the femoral artery. Gli1^+^ cells are critical adventitial progenitors in vascular remodeling after acute and during chronic injury [70]. Thus, Gli1^+^ adventitial cells play a critical role in vascular calcification in CKD.

## 5. Vitamin D Supplements Have Therapeutic Effects on Vascular Calcification in CKD (Table 1)

In addition to vitamin D-dependent vascular calcification because of excessive use of VDRAs, vitamin D deficiency is related to vascular calcification in CKD. The normal 25(OH)D level in the blood is 30–80 ng/mL (75–200 nmol/L). Although a consistent conclusion has yet to be reached, most professionals have reported levels of 20–30 ng/mL (50–75 nmol/L) as vitamin D deficiency [4]. Vitamin D supplements are typically prescribed at levels lower than 30 ng/mL (75 nmol/L) in patients with CKD; vitamin D deficiency is associated with higher mortality in these patients. Although the cutoff value of serum 25(OH)D and vascular calcification remains controversial, vitamin D deficiency is associated with vascular calcification in patients with CKD. In uremic vascular calcification, a low serum 25(OH)D level is related to more severe calcification in patients with CKD [71]. Luo et al. reported that, in patients with CKD not undergoing HD, a serum 25(OH)D level lower than 20 ng/mL was associated with increased arterial stiffness [72]. In patients with ESRD, 25(OH)D negatively correlated to the severity of coronary arterial calcification, and a lower serum 25(OH)D level is associated with aortic pulse velocity [59]. In an animal study, vitamin D deficiency accelerated vascular calcification and atherosclerosis, independent of the expression of LDL receptors on vessels [73]. In LDL-knockout mice, a low vitamin D diet stimulated the osteogenic expression of SMCs [74]. In uremic vascular calcification with vitamin D deficiency, vitamin D supplements exert a protective role.

### 5.1. Vitamin D Supplements for Endothelial Dysfunction

As stated in the previous section, multiple factors affect endothelial function during early-stage CKD. The expression of VDRs on endothelial cells remains controversial; therefore, we discuss the effects of vitamin D on hormone release.

#### 5.1.1. RAAS Inactivation

Vitamin D deficiency has been associated with systemic and intrarenal RAAS activation in humans after angiotensin II infusion [16]. Vitamin D analogues or endogenous vitamin D supplements in addition to RAAS blockade agents have been reported to exert additive effects for reducing proteinuria [75]. Active vitamin D downregulates renin expression by suppressing renin gene transcription [76]; it reduces urinary angiotensinogen and intrarenal RAAS blockade [77]. These observations reveal that vitamin D supplements reduce the activation of the RAAS system in patients with CKD, with or without angiotensin converting enzyme inhibitors or angiotensin-receptor blockers.

#### 5.1.2. Reduction of Cholesterol and Inhibition of Foam Cell and Cholesterol Efflux in Macrophages

The activation of VDRs by 1,25(OH)2D reduces liver and serum cholesterol levels because VDRs suppress the expression of the small heterodimer partner and activation of cholesterol 7-*α*-hydroxylase (CYP7A1). CYP7A1 is the rate-limiting enzyme in bile and reduces the serum cholesterol concentration [78, 79]. Oxidized LDL cholesterol retention in the vascular wall is harmful for the activation of immune cells, thus decreasing cholesterol efflux and releasing proinflammatory cytokines [80]. The differentiation of monocytes into M1 macrophages by interferon-*γ* is associated with higher endothelial stress and atherosclerotic plaque formation. Oh et al. reported that, in patients with diabetes mellitus, macrophages incubated with 1,25(OH)2D suppressed the formation of foamy cells by reducing acetylated or oxidized LDL cholesterol uptake [81]. Riek et al. reported that monocytes in patients with diabetes mellitus tend to differentiate to M2 macrophages on incubation with 1,25(OH)_2_ vitamin D_3_, and endoplasmic reticulum stress is alleviated [82]. Vitamin D supplements reduce hypertension and atherosclerotic changes in mice [83]. Thus, vitamin D plays a role in reducing the formation of atheromas or atherosclerotic changes.

#### 5.1.3. Vascular Regeneration

1,25(OH)2D directly influences VSMC regeneration through VDRs. Wu-Wong et al. reported that vitamin D downregulated thrombotic molecules from VSMCs from a human aortic cell culture. 1,25(OH)2D modified the vascular tone by regulating nitric oxide release from VSMCs [84]. Nutritional vitamin D supplements provide circulating CD45-negative and CD117-, stem cell antigen-1-, and fetal liver kinase 1-positive angiogenic myeloid cells, which are considered to promote vascular regeneration. 1,25(OH)2D promotes reendothelialization in injured endothelial cells by increasing stromal cell-derived factor, which is associated with the homing of angiogenic myeloid cells [85]. However, the interaction between progenitor cells of angiogenesis and supplements of vitamin D should modulate the vascular regeneration by affecting the interaction between endothelial cells and VSMCs.

### 5.2. Vitamin D Supplements for Arterial Calcification: Mechanisms

#### 5.2.1. Application in Renal Osteodystrophy

In high-turnover bone disease, PTH suppression depends on active or nutritional vitamin D supplements. Active vitamin D suppresses the chief cells of PTH, and several derivatives reduce vascular calcification through anti-inflammatory effects [86]. However, hypercalcemia and hyperphosphatemia are common, and they are associated with further vascular calcification. Vitamin D derivatives, such as paricalcitol, are associated with a reduced incidence of hypercalcemia and hyperphosphatemia and lower severity of vascular calcification [86, 87]. Nutritional vitamin D inhibits PTH in patients with CKD [88, 89] and is associated with a lower incidence of hypercalcemia than active vitamin D. Nutritional vitamin D is converted to 1,25(OH)2D in the parathyroid gland through autocrine or paracrine mechanisms, and it binds to 24-hydroxylase to avoid 1,25(OH)2D degradation [90]. In low turnover disease, both vitamin D analogues and nutritional vitamin D alleviate vascular calcification. Mathew et al. reported that, in mice with adynamic bone disease, vitamin D analogues restored osteoblast activity, increased the osteoid volume, and reduced intravascular calcium accumulation [91]. At physiological concentrations, osteoblasts are activated and bone formation is accelerated. Eldecalcitol [1*α*,25-dihydroxy-2*β*-(3-hydroxypropyloxy) vitamin D3] is an analogue of 1*α*,25-dihydroxyvitamin D3 [1,25(OH)2D3], bearing a hydroxypropyloxy residue at the 2*β* position. In preclinical studies, eldecalcitol suppressed bone resorption to a greater extent than alfacalcidol but had a similar effect on bone formation and calcium metabolism, resulting in a greater increase in bone mineral density in ovariectomized rats [92]. Because eldecalcitol stimulates intestinal calcium absorption and improves calcium balance in addition to its skeletal effects, combination treatment with antiresorptive agents may be able to show better effects than native vitamin D and calcium supplementation in preventing fractures in patients with high bone turnover bone disease. Furthermore, during the treatments, the patients should keep negative extraosseous calcium balance and minimize total positive calcium balance as possible.

Further studies are warranted to clarify these issues. Under controlled concentrations of calcium and phosphate, vascular calcification can be controlled by correcting renal osteodystrophy.

#### 5.2.2. Restoring Calcification Inhibitors

Vitamin D analogue supplements help to restore vascular calcification inhibitors. Hansen et al. reported that fetuin-A significantly increased in patients undergoing HD who were receiving alfacalcidol rather than paricalcitol [93]. In addition, in arteries of patients with CKD, VDRAs restored the mRNA expression of klotho. The local restoration of klotho reversed the anticalcifying effect of FGF23 [56]. Moreover, VDRAs increased the expression of osteopontin in aortic cells from uremic mice [94]. Cianciolo et al. reported that VDRAs reduced endothelial progenitor cells' expression of osteocalcin, which is a calcification promoter [95]. Therefore, vitamin D might restore calcification inhibitors in patients with uremia and alleviate vascular calcification.

From the perspective of endothelial dysfunction and arterial calcification during GFR decline, vitamin D supplements should provide protection against vascular calcification.

### 5.3. Effects of Vitamin D Supplements on Vascular Progenitor Cells: Vitamin D/Sonic Hedgehog Signaling and Gli1+ Cells in Vascular Calcification of CKD (Figure 2)

Studies using primary cultured human keratinocytes have reported that 1,25(OH)_2_-dihydroxyvitamin D_3_ suppresses cyclin D1 and Gli1. The blockade of VDR by siRNA resulted in the hyperproliferation of keratinocytes and increased expression of cyclin D1 and Gli1 [96]. In a murine breast cancer cell line, VDRs regulated specific microRNA and secreted the Hh pathway inhibitor SuFu, which suppresses breast cancer cell proliferation [97]. Hh signaling plays an essential role in tissue differentiation during embryogenesis and maintains stem cell populations in certain adult tissues. The potential mechanisms of cross-talk between Hh signaling and calcitriol-VDR signaling suggest a cooperative role during multiple stages of human development and diseases [98]. The most common target associated with the Hh pathway is* Gli1*, which controls the expression levels of multiple genes related to Hh signaling, cell cycle progression, cell adhesion, and apoptosis [99]. These findings suggest that vitamin D deficiency contributes to vascular calcification through increased* Gli1* expression in CKD.

## 6. Vitamin D Supplements for Vascular Calcification: Clinical Evidence

Vitamin D deficiency is common in CKD and is associated with vascular calcification; therefore, vitamin D supplements are considered. In early CKD with a preserved GFR, vitamin D supplements have additive effects in reducing blood pressure, without affecting calcium or phosphate concentrations. Clinical studies have reported an antiproteinuric effect of vitamin D supplements. The supplements result in efficient sugar control by reversing insulin resistance. In advanced CKD, active vitamin D provides control over PTH. In addition, retrospective studies have reported reduced cardiovascular mortality in patients undergoing maintenance HD who were receiving active vitamin D supplements [87, 100, 101]. Therefore, adequate vitamin D supplements have been used as a cardioprotective agent.

Because of its potential role in cardiovascular protection, vitamin D has been used in several clinical trials. The VITAL study initiated in 2008 reported the effects of VDRAs in patients with CKD. Furthermore, 24 weeks of paricalcitol at a dosage of 1-2 *μ*g/day alleviated proteinuria in patients with diabetic nephropathy. Serum alkaline phosphatase and intact PTH were reduced in patients receiving paricalcitol [102]. Thethi et al. reported that, in patients with diabetes mellitus having an eGFR between 15 and 59 mL/min/1.73 m^2^, a daily supplement of paricalcitol did not improve brachial artery flow-mediated dilatation or nitroglycerine-mediated dilation [103]. In two recent double-blind RCTs (PRIMO and OPERA studies) in nondialysis CKD stages 3–5 patients active vitamin D (paricalcitol) failed to demonstrate the improvement in clinically cardiac outcome but did demonstrate an increased risk of hypercalcemia [104, 105]. Active vitamin D analogues, particularly the nonselective forms, are associated with hypercalcemia and hyperphosphatemia because of a direct effect on the intestinal absorption of calcium and phosphate.

In contrast to active vitamin D or vitamin D analogues, nutritional vitamin D is associated with a lower incidence of hypercalcemia [106, 107], and it acts as the substrate of extrarenal 1-*α*-hydroxylase. Cholecalciferol at a dosage of 25,000 IU every 2 weeks was effective, without inducing hypercalcemia or hyperphosphatemia in patients undergoing HD [107]. For nutritional vitamin D supplementation, a six-month course of oral cholecalciferol treatment was given to adult (age: 53.8 ± 17.3) long-term maintenance dialysis patients with vitamin D insufficiency. The cholecalciferol replacement did not demonstrate an increased risk of hypercalcemia [108]. In the study of cardiovascular disease in young adults with childhood onset of ESRD, the Berlin pediatricians Briese et al. [109] pointed out interesting differences with a quite similar study reported by Heidelberg [4]: the prevalence of coronary calcifications (10 versus 92%) and of cardiac valve calcifications (0 versus 32%) was quite lower in the Berlin study (more use of cholecalciferol) than in the Heidelberg one (less use of cholecalciferol), while the technique of evaluation was comparable. These findings suggest that nutritional vit-D supplement may prevent the development of vascular calcification [110]. Notably, both high and low vitamin D levels are associated with vascular calcification, and supplementation with excessive or insufficient exogenous vitamin D has been associated with vascular calcification in in vivo and human studies [111]. Assimon et al. reported that ergocalciferol reduced the plasma concentration of adhesion molecules in patients undergoing HD [112]. Several clinical trials have analyzed the effects of nutritional vitamin D on patients with CKD and ESRD. Hewitt et al. [113] reported that cholecalciferol administered to patients with ESRD for more than 6 months reduced tartrate-resistant acid phosphatase-5b but not the pulse-wave velocity. Kidir et al. reported that cholecalciferol improved arterial diastolic function in patients undergoing dialysis [108]. Since the mechanism of vascular calcification in CKD is complex, early use of nutritional vit-D in CKD may be helpful although its therapeutic effect is still unproven.

## 7. Native Vitamin D Supplements for CKD Patients: The Timing for Supplement and the Dosage

### 7.1. Check Serum 25(OH)D Level Since CKD Stage 3

Under normal renal function, high or low serum 25(OH)D levels do not adequately affect 1,25(OH)_**2**_D levels. However, in advanced kidney diseases (glomerular filtration rate [GFR] < 25 mL/min), the serum 25(OH)D and 1,25(OH)_**2**_D levels exhibit strong relationships because diminished 25(OH)D can be converted to its bioactivated form 1,25(OH)_**2**_D by the residual renal 1-*α*-hydroxylase in CKD. Higher physiological 25(OH)D levels can upregulate serum 1,25(OH)_**2**_D levels in CKD patients [114]. Thus, the most appropriate time for initiation of therapy will be stages 3-4 of CKD with low serum 25(OH)D levels. Regular check serum levels of 25(OH)D will be the adequate therapeutic strategies.

### 7.2. The Advantage of Native Vitamin D in CKD

Compared with active vitamin D (calcitriol), 25-hydroxy vitamin D (calcifediol) has a longer plasma half-life and less potency, with fewer effects on hypercalcemia; thus, it has become a crucial agent for vitamin D replacement in CKD patients. A previous study of the pharmacokinetics of oral cholecalciferol and calcifediol revealed that calcifediol given daily, weekly, or as a single bolus is approximately 2-3 times more potent in increasing plasma 25 (OH) D3 concentrations than cholecalciferol is [36]. Recently, a modified-release oral formulation of calcifediol was designed to gradually raise serum 25-hydroxyvitamin D to minimize the induction of CYP24 (the cytochrome P-450 enzyme that specifically catabolizes vitamin D and its metabolites) and was found to reduce iPTH more effectively in patients with secondary hyperparathyroidism [115].

Native vitamin D supplementation prevents secondary hyperparathyroidism in early CKD; KDOQI suggests its use is not beneficial in advanced CKD because of the lack of 1-*α*-hydroxylase in the kidneys [116]. However, another study indicated that 1,25(OH)2D levels were increased after supplementation with native vitamin D in hemodialysis patients and suggested that there was enough extrarenal 1-*α*-hydroxylase activity to produce serum levels of 1,25(OH)2D even in ESRD [117]. We also had shown that cholecalciferol, in combination with paricalcitol, additively lowers the iPTH levels in a significant number of HD patients with SHPT. A dose of 5000 IU/week of cholecalciferol could maintain serum 25(OH)D3 levels above 30 ng/dL as early as 8 weeks after beginning supplementation [118]. The combination therapy of native vitamin D and active vitamin D supplements has fewer adverse effects of hypercalcemia and hyperphosphatemia and can improve bone quality efficiently. In patients with adynamic bone disorder (low bone turnover), the viability of osteoblasts and osteoclasts is low. Providing native vitamin D or intermittent PTH supplements may rescue the function of osteoblasts, improve bone turnover, and promote bone health.

### 7.3. The Dosage of Native Vitamin D in CKD Patients

Public-health authorities are responsible for ensuring the recommended daily vitamin D intake of 600–800 IU in the general population (under circumstances of limited or no sun exposure). With regard to patient care, the antifracture effects of vitamin D have been documented for daily vitamin D doses of 800–2,000 IU, whereas daily vitamin D doses of up to 4,000 IU (and probably even 10,000 IU) are considered to be safe with regard to acute vitamin D toxic effects leading to hypercalcemia [4].

Vitamin D supplementation to maintain 25-OHD concentrations at 20–30 ng/mL or higher (but <50 ng/mL) with or without VDRA therapy is inexpensive, appears safe, and may have additional health benefits in patients with > stage 3 CKD [119]. Daily vitamin D intake of 600–800 IU in CKD patients without high PTH levels is recommended. In CKD with high PTH levels, a minimum daily dose of 2,000 IU of vitamin D3 (equivalent to 14,000 IU/wk) likely is required to achieve serum 25(OH)D concentrations >30 ng/mL [120].

## 8. Conclusion

Vitamin D deficiency is common in patients with CKD because of GFR decline, renal tubular dysfunction, and proteinuria. With CKD progression, multiple factors exacerbate vascular calcification, including vitamin D deficiency. VDRAs or nutritional vitamin D supplements facilitate the alleviation of vitamin D-dependent or vitamin D-independent vascular calcification. Nonselective VDRAs may increase vascular calcification by inducing hyperphosphatemia and hypercalcemia. Nutritional vitamin D supplements may provide an ancillary role for ameliorating uremic vascular calcification.

## Figures and Tables

**Figure 1:**
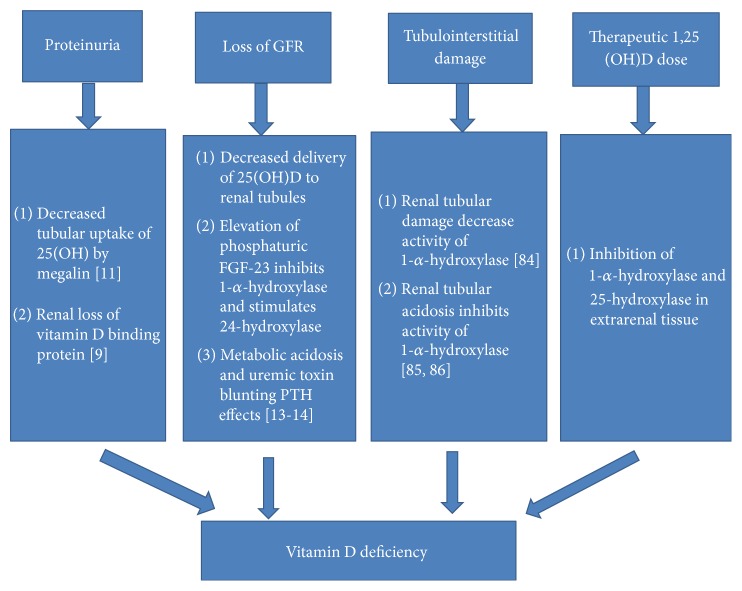
The factors inducing vitamin D deficiency in chronic kidney disease.

**Figure 2:**
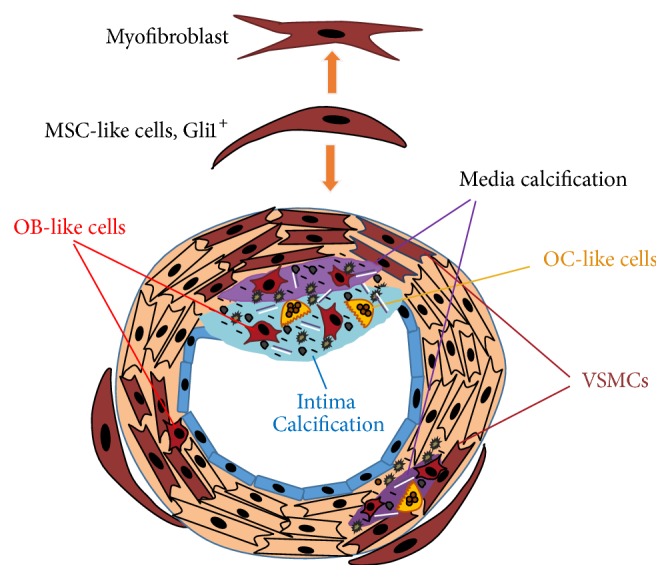
Vit-D deficiency may contribute to vascular calcification through increase in Gli1 expression in CKD. Mesenchymal stem cell- (MSC-) like cells reside in the vascular wall, especially in the inner layer of tunica adventitia. Perivascular Gli1+ progenitors are key contributors to injury-induced organ fibrosis. Gli1+ adventitial cells have a critical role in vascular calcification in CKD. 1,25(OH)_2_-dihydroxyvitamin D_3_ (1,25(OH)_2_D_3_) suppresses Gli1. Blockage of VDR by siRNA resulted in increased expression of Gli1. Vitamin D receptor regulates specific microRNA and secrets hedgehog (Hh) pathway inhibitors Suppressor of Fused (SuFu). The vit-D deficiency may contribute to vascular calcification through increase in Gli1 expression in CKD.

**Table 1:** Potential roles of vitamin D in preventing vascular calcification on endothelium and vascular smooth muscle.

Alleviating endothelial calcification	Mechanism	Alleviating arterial calcification	Mechanism
Inhibition of foam cell and cholesterol efflux in macrophage	(1) Activation of cholesterol 7-*α*-hydroxylase (2) Decrease of oxidative LDL uptake by foamy cells (3) Decrease of ER stress by differentiation of macrophage	Treatment on renal osteodystrophy	(1) On high-turnover osteodystrophy: inhibition of parathyroid hormone (2) On low-turnover osteodystrophy: restoring osteoblast activity

Enhancing vascular regeneration	(1) Providing circulating CD45−CD117+Sca1+Flk1+ angiogenic myeloid cells (2) Downregulating thrombotic cytokine from vascular smooth muscle cells	Restoring calcification inhibitors	(1) Increase of fetuin-A concentration (2) Restoring local klotho expression (3) Restoring osteopontin expression

RAAS system inactivation	(1) Downregulating renin expression (2) Decrease of urine angiotensinogen and intrarenal RAAS activation	Regressing residual proteinuria	(1) RAAS inactivation (2) Lessening TGF-*β*1 induced tubulointerstitial fibrosis

Improving insulin resistance	(1) Decrease of pancreatic beta cell destruction (2) Increase of beta cell insulin secretion (3) Increase of insulin sensitivity		
